# Localization and Upregulation of the Nasal Histamine H1 Receptor in Perennial Allergic Rhinitis

**DOI:** 10.1155/2012/951316

**Published:** 2012-10-23

**Authors:** Hideaki Shirasaki, Etsuko Kanaizumi, Nobuhiko Seki, Tetsuo Himi

**Affiliations:** Department of Otolaryngology, School of Medicine, Sapporo Medical University, S-1 W-16, Chu-ku, Sapporo 060-8543, Japan

## Abstract

In the present study, we have investigated the expression of histamine H1 receptor in human turbinates by RT-PCR, western blotting, and immunohistochemistry. Human turbinates were obtained by turbinectomy from 12 patients with nasal obstruction refractory to medical therapy. RT-PCR analysis of total RNA extracted from human nasal turbinate, primary cultured human nasal epithelial cells, and nasal vascular endothelial cells demonstrated the expression of histamine H1 receptor mRNA. About 56 kDa band was detected in human turbinates by western blot analysis using anti-H1 receptor antibody. The expression level of H1 receptor protein was marked in patients with nasal allergy than in patients with nonallergic rhinitis. The immunohistochemical study revealed that epithelial cells and vascular endothelial cells showed intense immunoreactivity for histamine H1 receptor. In addition, the blood vessels in superficial area expressed higher level of H1 receptor immunoreactivity than that in deeper area in the nasal mucosa. These results may have an important clinical implication for understanding the role of histamine H1 receptor on upper airway diseases such as allergic rhinitis and nonallergic rhinitis.

## 1. Introduction

 The allergic response is a complex process involving the interaction of many mediators. Histamine is the most important mediator in the pathogenesis of nasal allergy [1]. Administration of exogenous histamine into human nasal airway causes nasal obstruction, rhinorrhea, and sneezing [2]. These effects appear to be mediated by histamine H1 receptor because H1 receptor antagonists abolish histamine-induced nasal symptoms [3]. 

To understand the role of histamine on nasal allergy, the information about the localization of histamine H1 receptor is very important. However, limited numbers of studies have been reported.

The previous autoradiografic study using ^3^H-pyrilamine has demonstrated H1 receptor existed exclusively on the endothelium of vessels [4]. More recently, Sanico et al. found that not only vascular endothelial cells but also epithelial cells and nerves expressed histamine H1 receptor on human inferior turbinates by immunohistochemical studies [5].

Mucosal hyperreactivity to histamine can be observed in patients with perennial allergic rhinitis, suggesting upregulation of histamine H1 receptor may exist [5]. However, little is known about upregulation of H1 receptor protein in upper airway. 

In the present study, western blotting, immunohistochemistry, and RT-PCR analysis for histamine H1 receptor were performed to confirm both mRNA and protein expression of the H1 receptor in human nasal mucosa. 

## 2. Materials and Methods

### 2.1. Tissue Preparation

Human inferior turbinates were obtained after turbinectomy from 12 patients with nasal obstruction refractory to medical therapy. Informed consent was obtained from all patients and this study was approved by the ethics committee of Sapporo Medical University. All were nonsmokers, and 6 patients had perennial allergy against mites as defined by questionnaire and CAP test (Pharmacia, Uppsala, Sweden). All medications, including antibiotics, were prohibited for at least 3 weeks prior to the study. Demographic and clinical characteristics of the patients are summarized in Table 1. The nasal mucosal specimens were dissected from the cartilage, and (1) immediately frozen in liquid nitrogen and stored at −70°c for RNA and protein extraction for RT-PCR and western blotting, (2) placed into cold transfer medium (RPMI 1640 medium) for epithelial cell and vascular endothelial cell culture, and (3) fixed in 10% formalin for immunohistochemistry.

### 2.2. Human Nasal Vascular and Epithelial Cell Culture

#### 2.2.1. Vascular Endothelial Cell Culture

Human nasal vascular endothelial cells (HNVECs) were isolated from nasal inferior turbinates according to a previously described protocol [6] with minor modification. The nasal specimen was cut into 2-mm^2^ sections and enzymatically digested using 0.2% collagenase type IV solution (Sigma, St Louis, MO, USA) for 5 min at 37°C, washed with MCBD 131 medium (Sigma) containing 5% FCS and 2 ng/mL vascular endothelial growth factor (Invitrogen Co., Carlsbad, CA, USA), and placed in collagen type-I-coated 6-well culture plates (Sumitomo Bakelite Co. Ltd., Osaka, Japan). After 24 hrs, the medium and the tissue pieces were discarded, and the culture plate was washed twice to remove floating cells. Fresh medium MCBD 131 medium (Sigma) containing 5% FCS and 2 ng/mL vascular endothelial growth factor was added, and the cells were cultured in a 5% carbon dioxide humidified atmosphere at 37°C. The culture medium was changed at day 1 and every two days thereafter. Monolayer cell confluence was achieved after 7–10 days of culture. Morphologic observations using a phase contrast microscope showed the HNVECs consisted primarily of vascular endothelial cells. More than 95% of the HNVECs showed positive reactions for anti-human CD31 antibody (Dako, Denmark). HNECs grown to 80% confluency were used for RT-PCR analysis.

#### 2.2.2. Epithelial Cell Culture

Human nasal epithelial cells (HNECs) were isolated from human nasal mucosa specimens according to a previously described protocol [7]. Nasal specimens were rinsed 2-3 times with Ham's F-12 medium (Sigma) supplemented with antibiotics and incubated in 0.1% protease type XIV in Ham's F-12 medium for 16 hours at 4°C. After incubation 10% fetal bovine serum was added to neutralize protease activity and epithelial cells were detached by gentle agitation. Cell suspensions were filtered through a 60-mesh cell dissociation sieve (Sigma) and centrifuged at 500 g for 10 min at room temperature. The cell pellet was then resuspended in hormonally defined Ham's F-12 culture medium (Ham's HD). Cell suspensions were plated onto collagen type-I-coated 6-well culture plates (Sumitomo Bakelite Co. Ltd.) in Ham's HD medium and cultured in a 5% carbon dioxide humidified atmosphere at 37°C. The culture medium was changed at day 1 and every two days thereafter. Monolayer cell confluence was achieved after 6–8 days of culture. Morphologic observations using a phase contrast microscope showed the HNECs consisted primarily of epithelial cells. More than 95% of the HNECs showed positive reactions for anti-human cytokeratin antibody (Dako, Denmark). HNECs grown to 80% confluency were used for RT-PCR analysis.

### 2.3. RNA Extraction and RT-PCR Analysis

Total RNA from nasal mucosa was extracted using Trisol reagent (Invitrogen, Carlsbad, CA, USA). Some of RNA samples (1 *μ*g/*μ*L) were treated with DNase I (10 u/*μ*L) for 30 min to avoid DNA contamination following the second RNA extraction. RT-PCR was carried out by using a commercial One Step RNA PCR Kit (Takara Biomedicals, Tokyo, Japan). RNA (50 ng) was reverse-transcribed into cDNA by incubating with 5U AMV reverse transcriptase XL at 50°C for 30 min. After denaturation at 94°C for 2 min, cDNA were amplified by 40 cycles of PCR (94°C for 30 s, 60°C for 30 s, and 72°C for 1 min) with 5U of AMV-Optimized Taq DNA polymerase to amplify genes for H1 receptor. For the histamine H1 receptor, the forward primers (116–135): 5′-GGTCTAAGACAGAGTTGGTGCT-3′ and the reverse primers (593–612): 5′-ATCCTCTTCTGCAACCTGGTCA-3′ (497-bp fragment) were used [8]. PCR products were analyzed by agarose electrophoresis in 1.5% gel and visualized by ethidium bromide staining. 

### 2.4. Western Blot Analysis

Tissue samples were homogenised in T-PER Tissue Protein Extraction Reagent (Thermo Fisher Scientific Inc, Waltham, MA, USA), supplemented with the proteiase inhibitor cocktail (Sigma). And the protein extracts were purified by using PAGEprep Advance Kit (Thermo Fisher Scientific Inc.). 

Protein concentration in the homogenates was determined using BCA Protein Assay Kit (Thermo Fisher Scientific Inc.). Protein extraxts (20 ug protein each) were separatd on a 4–12% sodium dodecyl sulphate-(SDS-) polyacrylamide gel and blotted onto a polyvinylidene difluoride (PVDF) membrane (Invitrogen Corporation, Carlsbad, CA, USA). Membranes were then blocked and probed with rabbit anti-human H1 receptor polyclonal antibody against a peptide corresponding to cytoplasmic loop#3 (between transmembrane 5 and 6) of human histamine H1 receptor (Alpha Diagnostics International, San Antonio, TX, USA) or rabbit anti-human glyceraldehyde-3-phosphate dehydrogenase (GAPDH) polyclonal antibody (Santa Cruz Biotechnology Inc, Santa Cruz, CA, USA) as internal control. The bands were visualised using a WesternBreeeze chromogenic western blot immunodetection kit (Invitrogen). To verify band specificity, anti-H1 receptor antibody was also preincubated with a 100× molar excess of blocking peptides (Alpha Diagnostics International). 

The ratio of the expression of H1 receptor protein to beta-actin protein was evaluated by densitometry.

### 2.5. Immunohistochemistry

#### 2.5.1. Antibodies

For immunohistochemistry of histamine H1 receptor, rabbit anti-human H1 receptor polyclonal antibody (Alpha Diagnostics International) was used at 5 *μ*g/mL concentration. 

#### 2.5.2. Immunohistochemistry

Deparaffinized sections were initially incubated with 3% H_2_O_2_ in methanol for 30 min to quench endogenous peroxidase activity. After microwave treatment (10 minutes at 500 Watt in citrate buffer), the sections were incubated in blocking solution (10% normal goat serum in PBS) for 30 min before incubation in primary antibody. Then, the sections were incubated with anti-H1 receptor polyclonal antibody for overnight at 4°C, washed, and incubated for 30 minutes with EnVision+, Peroxidase, Rabbit (Dako Corporation, Carpinteria, CA, USA) which reacts with rabbit immunoglobulins. A further washing in PBS was followed by developing in DAB (Dako) as a chromogen for signal visualization. The slides were counterstained Mayer's haematoxylin and coverslipped using mounting medium. To verify the signal specificity, anti-H1 receptor antibody was also preincubated with 100× molar excess of blocking peptide (Alpha Diagnostics International). Negative controls were obtained by replacing primary antibodies by rabbit immunoglobulin fraction (Dako).

### 2.6. Statistical Analysis

Histamine H1 receptor and GAPDH protein levels evaluated by western blot analysis were determined in duplicate and data are presented as mean ± SD. The nonparametric Mann-Whitney *U* test was used to determine difference in between perennial allergic rhinitis and nonallergic rhinitis. Values with *P* ≤ 0.05 were considered to be statistically significant.

## 3. Results

### 3.1. RT-PCR Analysis

**Figure 1:**
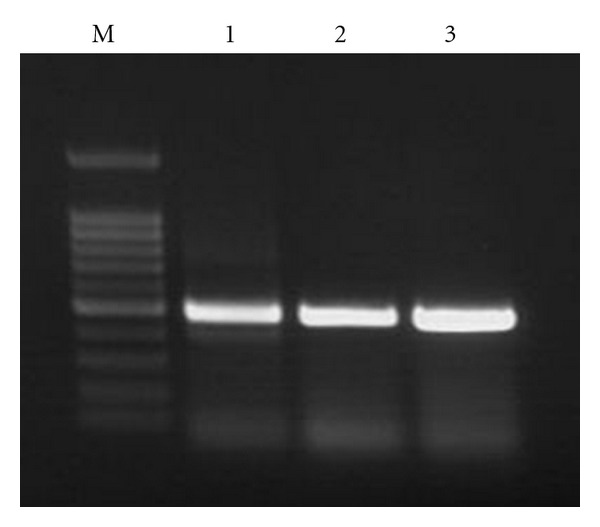
Detection of histamine H1 receptor mRNA by RT-PCR for 40 cycles of amplification from inferior turbinate, primary cultured human nasal epithelial cells, and primary cultured human vascular endothelial cells. RNA was extracted, RT-PCR performed using H1 receptor primers, demonstrating 497 bp fragment. Lane M: 100 bp ladder. Lane 1: PCR products from total RNA of human inferior turbinate. Lane 2: PCR products from total RNA of primary cultured human nasal epithelial cells. Lane 3: PCR products from total RNA of primary cultured human nasal vascular endothelial cells.

Figure 1 shows the results of RT-PCR for 40 cycles using total RNA extracted from human nasal mucosa (Figure 1, lane 1), primary cultured human nasal vascular endothelial cells (Figure 1, lane 2), and primary cultured human nasal epithelial cells (Figure 1, lane 3), which revealed the expression of histamine H1 receptor mRNA. These results suggested that the existence H1 receptor mRNA in human nasal epithelial cells and vascular endothelial cells.

### 3.2. Western Blotting

**Figure 2:**
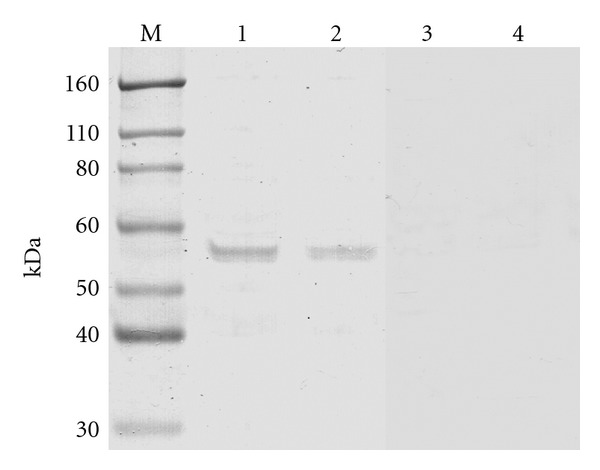
Representative western blots for the H1 receptor protein in human nasal mucosa (lane 1: perennial allergic nasal mucosa, lane 2: nonallergic nasal mucosa). The antibody recognized a single protein of 56 kDa referred as H1 receptor. In control experiments, anti-H1 receptor antibody was preincubated with specific blocking peptides (lane 3: control for lane 1, lane 4: control for lane 2).

In order to determine the protein size of H1 receptor in human nasal mucosa, H1 receptor expression was confirmed by using western blot analysis. As shown in Figure 2, the expression of single band for H1 receptor protein could be demonstrated (Figure 2, lane 1 and 2). The band of approximately 56 kDa is of the expected size [9]. The specificity of the polyclonal antibody was ascertained by complete neutralization through preincubation of the anti-H1 receptor polyclonal antibody with its respective specific blocking peptide immunogen (Figure 2, lanes 3 and 4).

**Figure 3:**
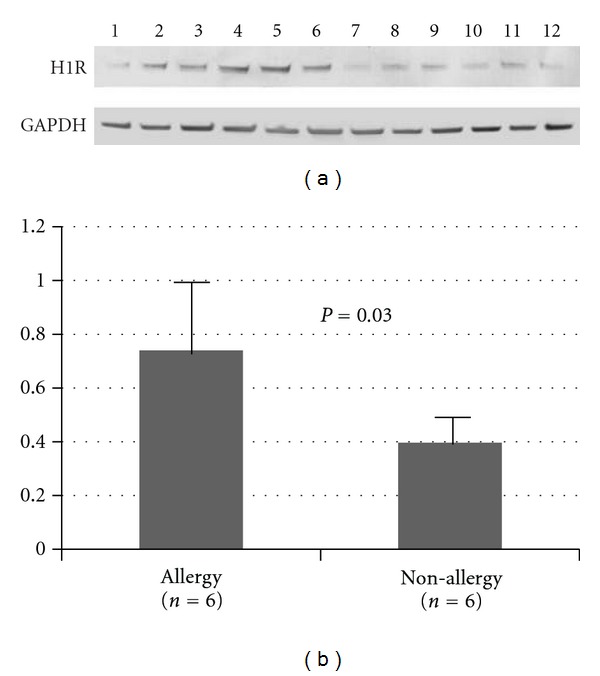
Western blots showing H1 receptor protein level in allergic nasal mucosa (lane 1–6) and nonallergic nasal mucosa (lane 7–12). Densitometric analysis of the relative protein level of H1 receptor as shown in (b). The data were expressed as mean ± SD and normalized to GAPDH.

Densitometric quantification of the bands was performed to evaluate the expression levels of H1 receptor on allergic and nonallergic nasal mucosa. The ratio of H1 receptor/GAPDH in nasal mucosa from patients with nasal allergy was significantly higher than the ratio found in nasal mucosa obtained from nonallergic rhinitis (allergic rhinitis: 0.76 ± 0.24, nonallergic rhinitis: 0.39 ± 0.11,  *P* = 0.03; Figure 3(b)).

### 3.3. Immunohistochemistry

**Figure 4:**
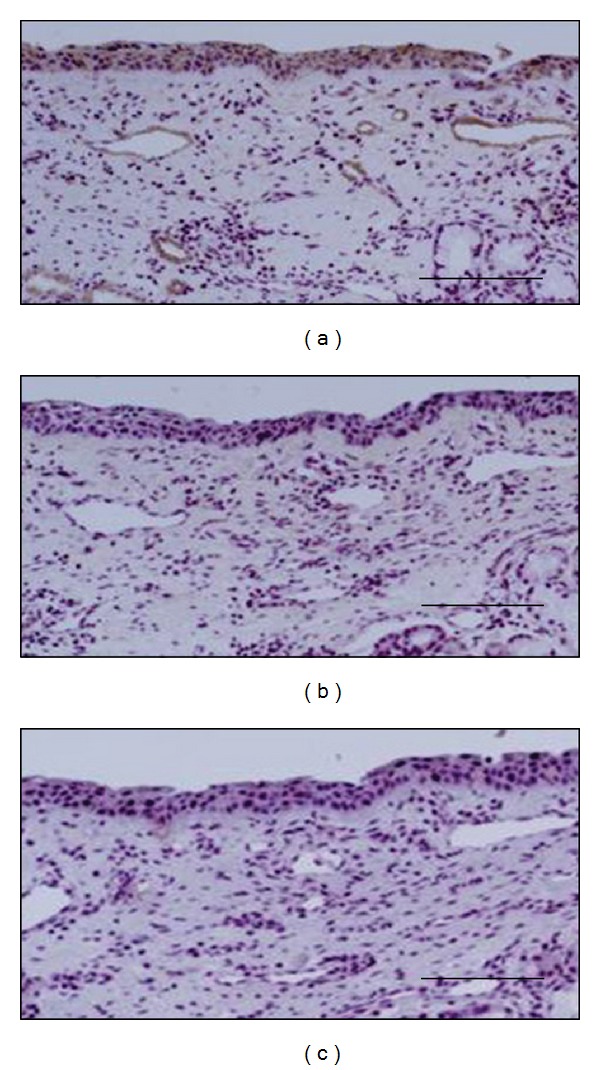
Immunohistochemical localization of histamine H1 receptor on human nasal mucosa. Inferior turbinates were stained with anti-human H1 receptor antibody (a, b) or normal rabbit immunoglobulin (c). The intensity of the H1 receptor staining was decreased by coincubation with a specific blocking peptide (b). Scale bar = 100 *μ*m.

**Figure 5:**
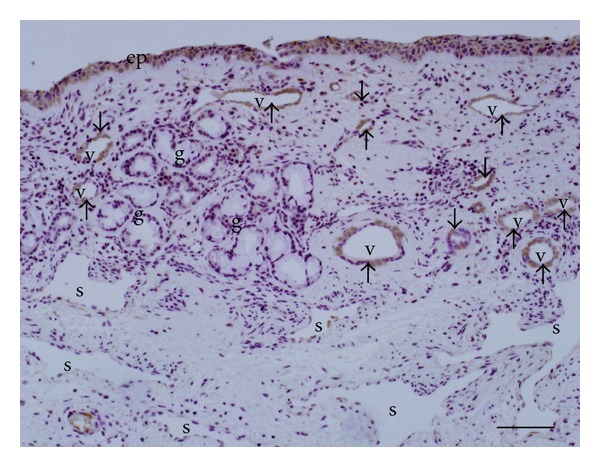
Immunohistochemical staining for histamine H1 receptor in human nasal mucosa. The arrows indicate the intense immunoreactivity of H1 receptor on vascular endothelial cells (V) in superficial region. In contrast, deep venous sinusoids (S) have little or no immunoreactivity for H1 receptor. (ep); airway epithelium, (g): submucosal glands. Scale bar = 50 *μ*m.

The distribution of H1 receptor in nasal mucosa was examined by means of immunohistochemistry. As shown in Figure 4(a), immunoreactivity for H1 receptor was significantly detected in epithelial cells and vascular endothelial cells. On the other hand, submucosal glands expressed little or no H1 receptor immunoreactivity. The intensity of the H1 receptor staining was decreased by coincubation with a specific commercial blocking peptide (Figure 4(b)). Specificity of the staining was also confirmed by the absence of labeling with normal rabbit immunoglobulin (Figure 4(c)). As shown in Figure 5, we found the heterogeneity of endothelial H1 receptor expression. The blood vessels in superficial area expressed higher level of H1 receptor immunoreactivity than that in deeper area in the nasal mucosa. 

## 4. Discussion 

The human H1 receptor is a 487-amino acid G-coupled protein with 7 transmembrane domains [9], and molecular mass of H1 receptor is calculated to be 55,781 Da. Thus, the molecular mass, about 56 kDa for the nasal H1 receptor estimated in this study is comparable with those noted in the previous reports.

Our present western blot analysis revealed that H1 receptor protein was significantly increased in allergic nasal mucosa in comparison with nonallergic nasal mucosa. Mucosal hyperresponsiveness is a pivotal pathophysiologic characteristic of allergic rhinitis. This phenomenon can be observed in patients with perennial allergic rhinitis, when compared with healthy control subjects [5]. It is therefore, logical to hypothesize that hyperresponsiveness might stem from upregulation of histamine receptors. 

In the past, only few studies have been designed to explore the expression of H1 receptor in nasal mucosa biopsies of patients with allergic rhinitis.

By using RT-PCR, H1 receptor mRNA levels have been found to be increased in nasal mucosa of perennial allergic rhinitis [10, 11], but not in seasonal allergic rhinitis [12]. On the other hand, little work has been done to evaluate the expression of nasal H1 receptor at protein level. Nakasaki et al., examining biopsy specimens from both humans and guinea pigs, could not demonstrate differences in H1 receptor density between allergic rhinitis and controls using the technique of radioligand binding. It is not yet clear why the levels of H1 receptor are elevated in allergic airway. However, several chemical mediators and cytokines including histamine, platelet activating factor [13], and IL-4 [14, 15] upregulate H1 receptor levels. So, our data implies that H1 receptor is upregulated in allergic nasal mucosa, playing a role in the pathogenesis in allergic rhinitis. 

 Our present immunohistochemical studies and RT-PCR analysis clearly show the expression of histamine H1 receptor on not only nasal vascular endothelial cell but also nasal epithelial cells at mRNA and protein level. In 1992, Okayama et al. demonstrated the presence of H1 receptors in the mucosa of human turbniates by using autoradiographic and pharmacologic techniques [4]. In their study, localization of H1 receptors was clear in the endothelium of arterioles, veins, and sinusoids. However, no evidence of binding to radioactive pyrilamine (mepyramine) could be generated on the nasal epithelium in their report [4]. As yet, several studies have reported the effects of histamine on airway epithelial cells in vitro. Histamine cause releases of cytokines and chemokines from cultured airway epithelial cells [16, 17]. In addition, histamine is an important mediator of epithelial barrier function, leading to increases in paracellular permeability [18, 19].

 The expression of H1 receptor protein was found not only epithelial cells, but also on superficial blood vessels, especially on endothelium of small vessels in lamina propria. H1 receptor stimulation induces various cellular responses in vascular endothelial cells. The increase in calcium induced by the activation of H1 receptors leads to the production of nitric oxide in vascular tissues, causing vasodilation, an endothelium- and cyclic guanosine monophosphate (cGMP)-dependent action [20]. The effects on vascular endothelial cells in vivo lead directly to changes in vascular permeability, allowing for leukocyte infiltration and oedema formation [21, 22]. In contrast, we could not confirm the H1 receptor expression on sinusoidal vessels, which are believed to regulate mucosal swelling in the nose [23]. Based on the location of H1 receptor, histamine may play minor role on the regulation of vascular swelling in nasal mucosa. This suggestion is supported by a small beneficial effect of H1 receptor antagonists on the nasal congestion during nasal allergic responses [24]. 

## 5. Conclusions

Using RT-PCR, western blot analysis, and immunohistochemistry, we have confirmed the expression and the distribution of histamin H1 receptor in human nasal mucosa. The expressions of H1 receptor protein were marked in patients with perennial nasal allergy than in patients with nonallergic rhinitis. Our findings should be of considerable interest for understanding the role of histamine in upper airway diseases such as allergic rhinitis and nonallergic rhinitis. 

## Figures and Tables

**Table 1:** Demographic characteristics of allergic and nonallergic patients.

	Allergic rhinitis *N* = 6	Nonallergic rhinitis *N* = 6
Sex (male/female)	2/4	3/3
Age	31 (19–58)	39 (28–55)
Specific IgE to house dust mite (d1) (kU/L)	2.7 (1.0–13)	<0.35
Total IgE (kU/L)	210 (10–387)	110 (10–185)
Blood eosinophils (cells/*μ*L)	370 (70–690)	135 (55–240)
Current nasal symptoms (number of patients)		
Nasal obstruction	6 (all patients)	4 (all patients)
Sneezing	4	0
Rhinorrhea	3	2

Data expressed as median values and range (in brackets).
